# Fine-grained alignment of cryo-electron subtomograms based on MPI parallel optimization

**DOI:** 10.1186/s12859-019-3003-2

**Published:** 2019-08-28

**Authors:** Yongchun Lü, Xiangrui Zeng, Xiaofang Zhao, Shirui Li, Hua Li, Xin Gao, Min Xu

**Affiliations:** 1 University of Chinese Academy of Sciences, Beijing, China; 2 Institute of Computing Technology of the Chinese Academy of Sciences, Beijing, China; 3 Key Laboratory of Intelligent Information Processing, CAS, Beijing, China; 4 Computational Biology Department, School of Computer Science, Carnegie Mellon University, Pittsburgh, USA; 5 King Abdullah University of Science and Technology (KAUST), Computational Bioscience Research Center (CBRC), Computer, Electrical and Mathematical Sciences and Engineering (CEMSE) Division, Thuwal, Saudi Arabia

**Keywords:** Stochastic average gradient, Fine-grained alignment, Cryo-ET, MPI

## Abstract

**Background:**

Cryo-electron tomography (Cryo-ET) is an imaging technique used to generate three-dimensional structures of cellular macromolecule complexes in their native environment. Due to developing cryo-electron microscopy technology, the image quality of three-dimensional reconstruction of cryo-electron tomography has greatly improved.

However, cryo-ET images are characterized by low resolution, partial data loss and low signal-to-noise ratio (SNR). In order to tackle these challenges and improve resolution, a large number of subtomograms containing the same structure needs to be aligned and averaged. Existing methods for refining and aligning subtomograms are still highly time-consuming, requiring many computationally intensive processing steps (i.e. the rotations and translations of subtomograms in three-dimensional space).

**Results:**

In this article, we propose a Stochastic Average Gradient (SAG) fine-grained alignment method for optimizing the sum of dissimilarity measure in real space. We introduce a Message Passing Interface (MPI) parallel programming model in order to explore further speedup.

**Conclusions:**

We compare our stochastic average gradient fine-grained alignment algorithm with two baseline methods, high-precision alignment and fast alignment. Our SAG fine-grained alignment algorithm is much faster than the two baseline methods. Results on simulated data of GroEL from the Protein Data Bank (PDB ID:1KP8) showed that our parallel SAG-based fine-grained alignment method could achieve close-to-optimal rigid transformations with higher precision than both high-precision alignment and fast alignment at a low SNR (SNR=0.003) with tilt angle range ±60^∘^ or ±40^∘^. For the experimental subtomograms data structures of GroEL and GroEL/GroES complexes, our parallel SAG-based fine-grained alignment can achieve higher precision and fewer iterations to converge than the two baseline methods.

## Background

Cryo-electron tomography visualizes the three-dimensional structures in situ and sub-molecular resolution within single cells [1–5]. However, due to the radiation damage caused by electrons, the original tomograms’ signal-to-noise ratio (SNR) is extremely small, which typically limits the resolution of the original tomograms data to 5-10 nm [6]. At such a low SNR, the traditional 3D image registration methods are very difficult to apply. In normal conditions, thousands or even tens of thousands of subtomograms are aligned and averaged to obtain structures with higher resolutions, which reduces noise and eliminates missing wedge effects. A number of individual macromolecules are picked from a 3D tomogram and then classified into structural classes by pair-wise comparisons. Alignment and averaging of subtomograms in each class result in a clearer structure with increased SNR.

Subtomogram alignment aims to rotate and translate a subtomogram to minimize its dissimilarity measure with a reference structure. The reference-free averaging process iteratively aligns a large number of subtomograms together with their own simple average as the initial reference to approximate the macromolecular structure of interest [7–10]. In the iteration procedure of optimizing subtomogram averaging, each subtomogram is rotated and translated in different ways but with the same reference structure. Much software has been developed for subtomogram alignment and classification [8, 11, 12]. Most implement algorithms that use a dissimilarity measure or a distance function as the alignment metric between the subtomogram and the reference [8, 12–14]. In three dimensional space, there is one translation and one rotation parameter along each axis. Therefore, for averaging *N* subtomograms, the parameter search space is 6^*N*−1^ dimensional. If an exhaustive 6D search was performed in Cartesian space or in Fourier space for each subtomogram, the computational cost would be infeasible. To accelerate the search of translational parameters, Fourier transform is commonly used [15]. However, the computational cost for the exhaustive search of rotational parameters is still a major bottleneck. Fast translation-invariant rotational matching that obtains better rotational parameter candidate sets using spherical harmonics functions in Fourier space [16] has been proposed [17, 18] and extended to subtomogram alignment [9, 10, 19, 20].

A local fine-grained alignment can be applied for obtaining a better rotational parameter candidate set close to the optimal solution. Based on previous local refinement alignment on a very sparsely distributed starting rotational parameter candidate set [20, 21], we further explore the potential of utilizing locally optimized alignment methods in a sparse rotational parameter candidate set.

In this article, we design a competent stochastic average gradient (SAG) fine-grained alignment algorithm for dissimilarity measure between a pair of subtomograms in real space. We utilize an MPI parallel architecture, which can distinctly fulfill the simultaneous improvement of different alignment candidates. We demonstrate our SAG-based fine-grained alignment algorithm on realistically simulated data of GroEL and experimental GroEL and GroEL/GroES complexes subtomograms. The results show that SAG-based fine-grained alignment method can achieve higher alignment precision and better averaging of subtomograms at a low SNR of 0.003 with tilt angle range from +60^∘^ to −60^∘^ and from +40^∘^ to −40^∘^, as compared to baseline methods.

## Methods

We design a three-dimensional fine-grained alignment framework for subtomogram alignment based on stochastic average gradient [22], which minimizes the dissimilarity score defined by the Euclidean distance between a function with fixed parameters and a function with optimized parameters. We design dissimilarity scores of subtomogram alignment with missing wedge correction: constrained dissimilarity score in real space. We provide parallelization of our algorithm on the MPI parallel computing platform.

### Parameter definitions

We define a subtomogram as an integrable function, $V(\mathbf {x}) \colon \mathbb {R}^{3} \ \rightarrow \ \mathbb {R}$. We define $\mathbb {T}_{T}$ as the operator of translation on subtomogram for $T \ \in \ \mathbb {R}^{3}$, which be expressed by

1 $$\begin{array}{@{}rcl@{}} \mathbb{T}_{T}V(\mathbf{x})\colon= V(\mathbf{x}-T) \end{array} $$

In the 3D rotation group *SO*(3), we define *Λ*_*R*_ as the operator of rotation for a rotation *R*, which be expressed by

2 $$\begin{array}{@{}rcl@{}} \Lambda_{R}V(\mathbf{x})\colon=V[R^{-1}(\mathbf{x})] \end{array} $$

where rotation *R* is a 3×3 rotation matrix [17]. The 3D subtomograms *V*(**x**) rotation and translation operation can be described as:

3 $$\begin{array}{@{}rcl@{}} \mathbb{T}_{T}(\Lambda_{R}V(\mathbf{x}))=V(R^{-1}(\mathbf{x}) - T) \end{array} $$

The transformation parameters include rotation operation and translation operation can be represent as $\beta =(R,T)=(\phi,\theta,\psi,\tau _{1},\tau _{2},\tau _{3})^{\intercal }$, where rotation parameters $R = {(\phi,\theta,\psi)}^{\intercal }$ can be deemed as Euler angles in the ‘*ZYZ*’ usage [23] or ‘*y*’ usage [24], and translation parameters as $T = (\tau _{1},\tau _{2},\tau _{3})^{\intercal }$.

### Fine-grained alignment of subtomograms using constrained dissimilarity measure in a real space

We now propose a fine-grained registration algorithm for the subtomogram alignment based on the stochastic average gradient. The goal of fine-grained alignment is to search for a local minimum value provided the given rough parameters of rotation *R* and translation *T*. To perform the alignment, one must define an alignment metric. We use a dissimilarity measure function for the alignment of two subtomograms. Many challenges exist, such as low resolution, low SNR, distortions owing to partial data loss (i.e., missing wedge effect). These factors must be considered during the subtomogram alignment procedure.

To handle the significant missing wedge in Fourier space, the most common approach to correct the missing wedge is the constrained correlation coefficient (CCC) measure recommended by Förster et al. [8]. A binary mask function $\mathcal {M} \ \colon \ \mathbb {R}^{3} \ \rightarrow \ \{0,1\}$ is defined to represent the corresponding missing wedge. In cryo-electron tomography with single tilt ±*θ*, the missing wedge mask functions $\mathcal {M}(\zeta) \colon = I_{(|\zeta _{3}|\leq |\zeta _{1}|tan(\theta))}(\zeta)$, where *I* is symbolic function [19]. The overlap region after the alignment of two subtomograms in the Fourier space $\Omega \ \colon =\mathcal {M}\Lambda _{R}\mathcal {M}$. It only considers the best overlap region by rotation in Fourier space when two subtomograms are aligned, and eliminates the transform depending on the property of Fourier space. To reduce the effects of noise, focus on the particles, we also define a binary mask *M* in real space.

Related to the Fourier space, the constrained function of subtomogram *f* can be expressed as:

4 $$\begin{array}{@{}rcl@{}} f^{\star}\colon=\frac{(FT^{-1}(FT(f) \cdot \Omega)-\bar{f^{\star}}) \cdot M(x,y,z)}{\sqrt[]{\sum_{x,y,z}((FT^{-1}(FT(f) \cdot \Omega)-\bar{f^{\star}}) \cdot (M(x,y,z)))^{2}}} \end{array} $$

where FT denotes the Fourier transformation, FT ^−1^ denotes the inverse Fourier transformation.

The subtomogram mean value of $\bar {f^{\star }}$ must be restricted to *M* and *Ω* :

5 $$\begin{array}{@{}rcl@{}} \bar{f^{\star}}\colon=\frac{1}{\sum_{x,y,z}M}\sum\limits_{x,y,z}FT^{-1}(FT(f) \cdot \Omega) \end{array} $$

The constrained function of subtomogram *g* can be expressed as:

6 $$\begin{array}{@{}rcl@{}} {g^{\star}_{\beta}\colon=\frac{(FT^{-1}(FT(\mathbb{T}_{T}{\Lambda_{R}}g) \cdot \Omega)-\bar{g^{\star}_{\beta}}) \cdot M(x,y,z)}{\sqrt[]{\sum_{x,y,z}((FT^{-1}(FT(\mathbb{T}_{T}{\Lambda_{R}}g) \cdot \Omega)-\bar{g^{\star}_{\beta}}) \cdot (M(x,y,z)))^{2}}}} \end{array} $$

where $\bar {g^{\star }_{\beta }} \colon = \frac {1}{\sum _{x,y,z}M}\sum _{x,y,z}FT^{-1}(FT(\mathbb {T}_{T}{\Lambda _{R}}g) \cdot \Omega)$.

In fact, for convenient calculation on discrete voxel points, we define the constrained cross-correlation function of normalized and aligned subtomograms *f*^⋆^ and $g^{\star }_{\beta }$ can be given as:

7 $$\begin{array}{@{}rcl@{}} CCC\colon=\sum\limits_{x,y,z}f^{\star}(x,y,z) \cdot g^{\star}_{\beta}(x,y,z) \end{array} $$

During the alignment, the dissimilarity score *d* is normalized, which is derived from the CCC. Given a normalized and aligned subtomogram *f*^⋆^ and $g^{\star }_{\beta }$, *d* can be represented as:

8 $$\begin{array}{@{}rcl@{}} d(f^{\star},g^{\star}_{\beta})\colon=(f^{\star}-g^{\star}_{\beta})^{2}=2-2{\cdot}CCC(f^{\star} \cdot g^{\star}_{\beta}) \end{array} $$

By using the fast rotational matching (FRM) [9, 19, 20], we can get an initial set of the top *N* best rough rotations candidate set { *R*^1^,*R*^2^,…,*R*^*N*^}, and then obtain the top *N* best rough translations candidate set { *T*^1^,*T*^2^,…,*T*^*N*^}, that can efficiently minimize the normalized Euclidean distance *d* using fast translational matching (FTM), where *N* is the cardinality of the rotations or translations set. The selected rotation candidate sets have the highest CCC value compared to other rotation sets that are not selected. For each rotation *R*^*j*^ in the set { *R*^1^,*R*^2^,…,*R*^*N*^}, we can utilize FTM to search the best translations *T*^*j*^ between *f*^⋆^ and $g^{\star }_{(T,R)}$. For comparison purpose, the acquisition of the initial rotations candidate set was implemented using the corresponding fast rotation matching code of Chen’s method. Two volume (subtomogram and reference) are transferred into Fourier space, the power spectrum (i.e. the magnitude of Fourier components) of a subtomogram and reference are only considered, and then we convert the Fourier coefficients to spherical coordinates and calculate fast rotational match by spherical harmonics convolution. The power spectrum is translation invariant. Therefore the fast rotation matching does not depend on translation.

Given a certain combination of *R* and *T*, we can get the new rotation value *R*^*k*^ and translation value *T*^*k*^ using the stochastic average gradient (SAG) fine-grained alignment algorithm on three-dimensional density map, so that the normalized Euclidean distance decreases.

9 $$\begin{array}{@{}rcl@{}} d_{R^{k},T^{k}} \ \geq \ d_{R^{k+1},T^{k+1}} \end{array} $$

The SAG algorithm was firstly applied to the two-dimensional matrix [22]. Standard stochastic gradient descent algorithm implements sublinear rates, because the randomness introduces variance. The SAG algorithm stores previous calculated gradients to achieve a linear convergence rate. We expand the SAG algorithm and apply it to the three-dimensional matrix to form the 3D SAG algorithm. We design a 3D version of SAG algorithm and apply it to 3D rigid registration on subtomogram alignment procedure.

Since the function *f*^⋆^ is fixed, we only use SAG fine-grained alignment algorithm to update the *β*=(*R*,*T*). Now we redefine the loss function *J* for 3D subtomogram alignment.

10 $$\begin{array}{@{}rcl@{}} J(\beta)=J(R,T) = \frac{1}{2n}\sum\limits_{i=1}^{n}h_{(R,T)}(x_{i}) \end{array} $$

where *n* is the length of the volume on the x-axis, *x*_*i*_ is a slice of subtomogram along x-axis, index *i* ∈ {1,..,n}, $h_{\beta }(x_{i}) = h_{(R,T)}(x_{i}) \ \colon = (f^{\star }(x_{i}) - g^{\star }_{(T,R)}(x_{i}))^{2}$.

The recursive form of the SAG algorithm is given as:

11 $$\begin{array}{@{}rcl@{}} \beta^{k} \ \colon= \beta^{k-1} - \frac{\alpha_{k}}{n}\sum\limits_{i=1}^{n}y_{i}^{k}, & k \geq 1 \end{array} $$

where at each iteration a index *i*_*k*_ along the x-axis in the experimental data is random selected redundantly and uniformly in {1,…,*n*}, *α*_*k*_ is step size and $y_{i}^{k}$ can be given as:

12 $$\begin{array}{@{}rcl@{}} y_{i}^{k}= \left\{ \begin{array}{l r} {h_{(R,T)}(x_{i})}^{\prime} & if \ {i=i_{k}} \\ y_{i}^{k-1} & otherwise \end{array} \right. \end{array} $$

Similar to the standard full gradient (FG) method, the procedure contains a gradient in regard to the whole experimental subtomogram data. However, similar to the stochastic gradient (SG) method, the each iteration of SAG method only calculates the gradient in regard to a slice of the whole experimental subtomogram data along the x-axis. So, the iterative cost is independent of *n*, thus giving the SAG method low iteration cost and a linear convergence rate. In other words, by randomly choosing index *i*_*k*_ and maintaining the memory of the latest gradient value calculated for each slice of the whole experimental subtomogram data, the iteration accomplishes a faster convergence rate than the iteration of the SG method. So SAG method does not increase the capability of getting trapped into local minima.

For our loss function *J*, we adopt empirical step size *α*_*k*_=1/*L*. In practice, Lipschitz constant *L* is unknown. The estimation of Lipschitz constant *L* will be doubled when the instantiated Lipschitz obeys the inequality [22].

We modify the estimation rule of Lipschitz constant *L* by selecting the max value in the experimental data.

13 $$\begin{array}{@{}rcl@{}} L^{i} = \lambda + A_{i} \qquad (Lipschitz \: constant \: for \: all \: J_{i}^{\prime}) \end{array} $$

where *A*_*i*_ denotes the one dimensional norm of maximum squared 3D matrix max*i*{∥*x*_*i*_∥^2^}.

We implement the method in Algorithm 1 through equation 11 and 12, and we utilize a variable *D* to express the gradient of *β*. For the purpose of parallelism and vectorization, the stochastic average gradient completions usually divide the data into “small batches” and implement the stochastic average gradient iterations on small batches. We similarly perform the 3D version of the SAG-based fine-grained subtomogram alignment on small batches (a slice) along the x-axis.


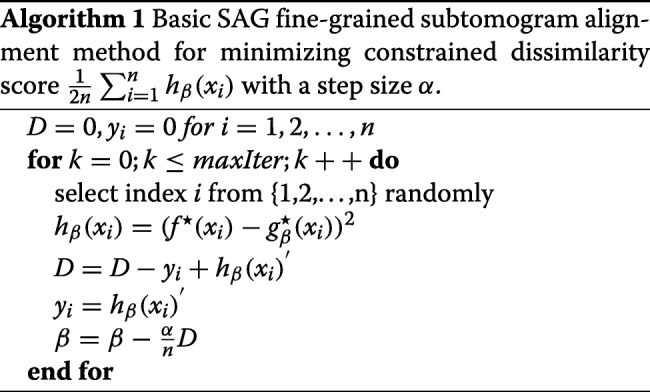


In order to speed up the SAG algorithm convergence rate and adequately decrease the memory space of SAG method, we optimize small batches SAG algorithm in 3D space, which select small batches slices along the x-axis in the experimental subtomograms data, rather than only selecting a slice along the x-axis in the experimental subtomograms data in Algorithm 2. In an optimized SAG fine-grained subtomogram alignment algorithm (Algorithm 2), small batches slices depends on the side length of subtomogram data, for example, small batches is about 4 ∼30 for our simulation subtomogram, in which the side length is 64. We use a loop to judge whether each slice is visited, instead of the visitation policy of each slice in the SAG algorithm.


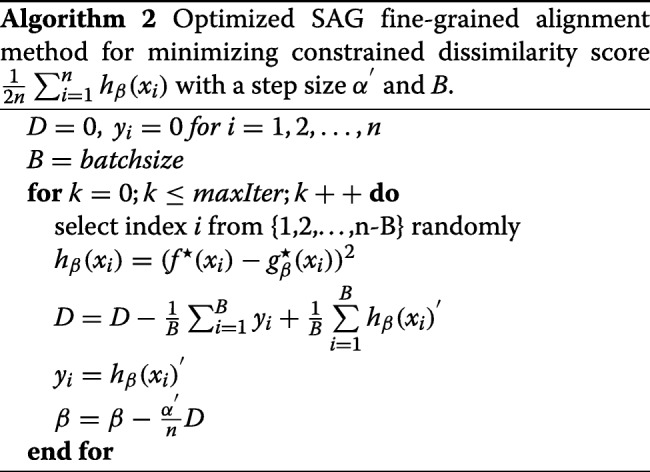


The comparison of computing time between Algorithm 1 and 2 is described in the Results section. Algorithm 2 is faster than Algorithm 1, so Algorithm 2 is selected for fine-grained subtomogram alignment. In the optimized SAG fine-grained subtomogram alignment algorithm, the number of x-slices in each iteration is about $\frac {1}{16}$ to $\frac {1}{2}$ of side length of subtomogram.

For the original candidate set *R* and *T*, the final result of iteration produces the refined parameters of subtomogram alignment $R^{k+1} = R^{k} -\frac {\alpha _{k}}{n}\sum _{i=1}^{n}y_{i}^{k}$ and $T^{k+1} = T^{k} -\frac {\alpha _{k}}{n}\sum _{i=1}^{n}y_{i}^{k}$ through optimized SAG fine-grained subtomogram alignment algorithm (Algorithm 2), where *k* and *k*+1 are the iteration numbers.

### Message passing interface frame parallel fine-grained subtomogram alignment procedure

To find global optimal rotation and translation parameters, it is necessary to perform multiple refining processes from different rotation and translation parameter candidate sets. To initialize on different parameter sets synchronously, we use Message Passing Interface (MPI) frame to calculate the score of dissimilarity in parallel. We compare dissimilarity scores gained by using different candidate rotation and translation parameter sets to find the least dissimilarity score in Algorithm 3. With the MPI parallel model, we can quickly search for the optimal rotation and translation candidate parameter in all candidate sets.


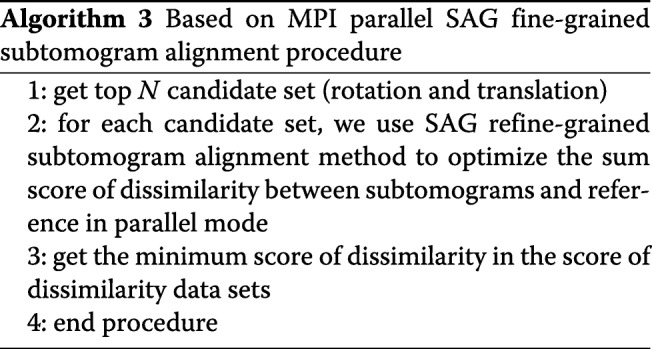


**Fig. 1:**
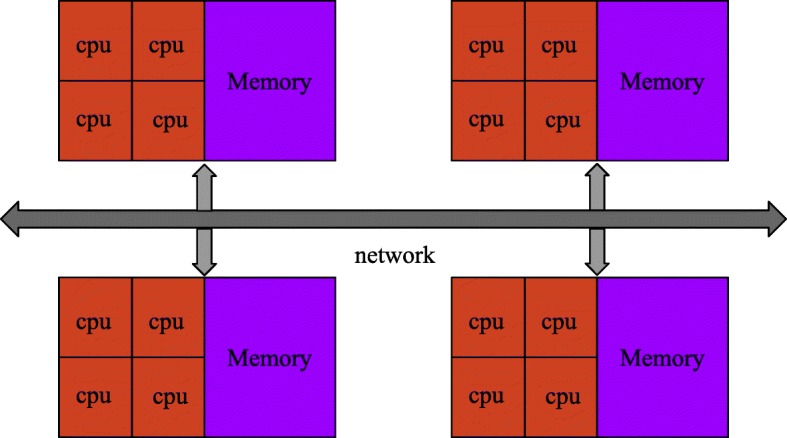
MPI architecture with different hardware platform

Message Passing Interface is a communication protocol on different computing nodes for concurrent computation, and supports peer to peer and broadcast. MPI is also a messaging application interface that includes protocol and semantic descriptions. MPI is specifically designed to allow applications to run in parallel on multiple independent computers connected over a network in Fig. 1.

We choose MPI frame as parallel programming for several advantages:

∙ MPI is the message passing library that can be regarded as a standard library. In fact, almost all HPC platforms support it.

∙ When we change applications to different platforms that conform to MPI standards, there is little or no need to modify the source code.

∙ There are many functions and a variety of implementations are available.

Finally, we outline some key differences of our stochastic average gradient fine-grained alignment method for the subtomogram alignment from Chen’s approach [20] and Xu’s approach [21]:

1. In Xu’s approach, they use Levenberg-Marquardt algorithm to calculate increment value, which needs total volume data to calculate the Jacobian matrix and parameters. In Chen’s approach, they calculate the cross-correlation coefficient of a 3D matrix in each iteration and find the best rotation and location values in the 3D matrix. They also utilize spherical harmonic function to calculate the new cross-correlation coefficient between the 3D experimental volume and the reference volume, to find the best cross-correlation score in each iteration.

2. Xu’s approach uses stochastic parallel refinement framework. Chen’s approach uses MPI frame to parallelize subtomogram alignment.

3. Our method utilizes a 3D version of stochastic average gradient algorithm to execute fine-grained subtomogram alignment and apply MPI frame to parallelize subtomogram alignment. Our SAG-based fine-grained alignment only needs a partial batch slices of the 3D volume in each iteration.

### Generating simulated cryo-electron tomograms

We downloaded the atomic model from Protein Data Bank (PDB), specified the resolution and voxel spacing, and conducted low-pass filtering of the data. After getting the density maps, we performed random rotation and translation operations. Contrast Transfer Function (CTF) was simulated using a known defocus value. The volume density maps were projected onto the specified tilt angles and angle increment. The projection images were applied with Gaussian-distributed noise and Modulation Transfer Function noise (MTF) to simulate electron optical effect. The projection images were reconstructed with a weighted back projection (WBP) algorithm to produce the simulated subtomogram datasets.

Atomic model (PDB ID:1KP8) was used to generate subtomograms of size 64^3^ with voxel size 0.6nm and -6 *μ*m defocus. We utilized tilt angle ±60^∘^ and ±40^∘^ with 1^∘^ angular increment respectively. The simulations procedure were implemented using the Situs PDB2VOL [25] program to get volume electron density maps.

**Fig. 2:**
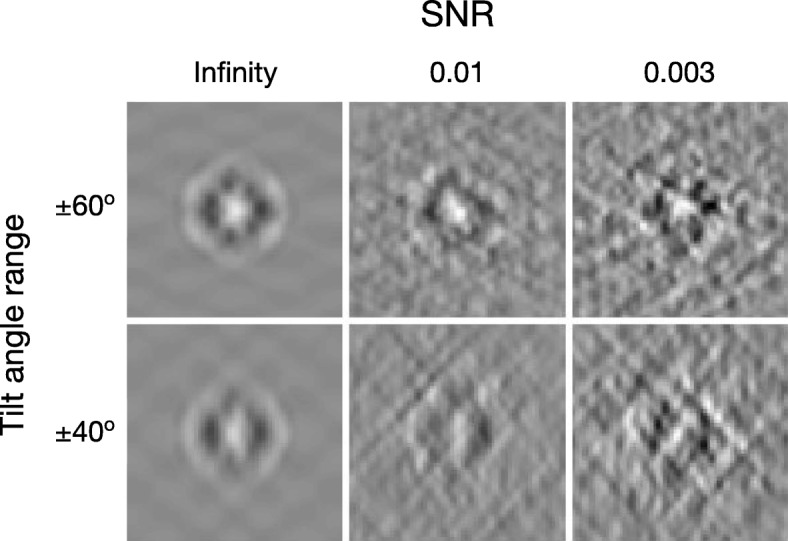
Center slices (x-z plane) of simulated subtomograms. Center slices (x-z plane) of simulated subtomograms (GroEL, PDB ID: 1KP8) of designated SNRs and tilt angle ranges

The central slices of different tilt ranges and SNRs are shown in Fig. 2. Subtomograms with smaller tilt range and lower SNR shows more deformation than noise-free subtomograms (i.e. reference).

### Experimental groEL and groEL/ES subtomograms

The experimental GroEL and GroEL/ES dataset were obtained in [8]. To collect the GroEL_14_GroES_7_, 1 *μ*M GroEL_14_ and 5 *μ*M GroES_7_ were incubated in a buffer for 15 min at 30^∘^*C*, which contained 5mM MgCl_2_, 5mM KCl, 5 mM ADP, 1mM DTT, and 12.5 mM Hepes (pH 7.5). 3.5 *μ*l of protein solutions were confused with 0.5 *μ*l of a 10 nm BSA-colloidal gold suspension using mesh grids. The sample was vitrified with plunge-freezing. The single-axis tilt series were obtained by a Tecnai G2 Polara microscope, which was equipped with 2k ×2k FEI CCD camera. The tilt series were acquired from tilt angle ±65^∘^ with 2^∘^ or 2.5^∘^ angular increment at a different defocus levels between 7 and 4 *μ*m. The object pixel size was 0.6nm.

## Results

### Classification of experimental groEL and groEL/ES subtomograms

Thousands of subtomograms, which also contain putative particles, were selected manually and aligned to subtomograms average according to cross-correlation. Eliminating lower cross-correlation coefficients (e.g., CCC ≤0.42), the remainder of particles were chosen for subtomogram alignment and classification. The dataset of experimental ∼800kDa GroEL_14_ and GroEL_14_/GroES_7_ subtomograms complex basically conducted as a quasi-standard in the subtomogram alignment and classification’s research [8, 12, 26, 27].

**Fig. 3:**
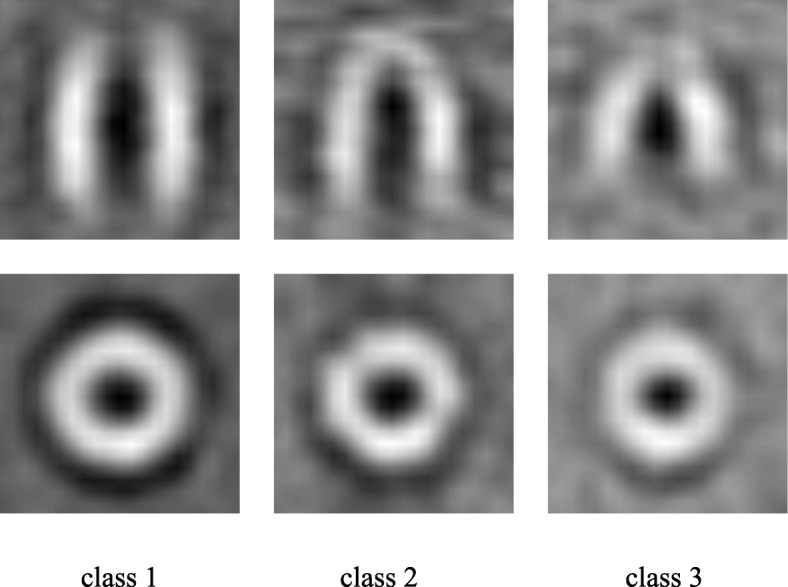
MCO-A classification of GroEL_14_/GroEL_14_GroES_7_ subtomograms complex. Slices of the three classes from MCO-A classification

The 786 subtomograms in the data set were aligned by the average of all subtomograms in the facultative direction and an unsupervised manner. Subsequently, we used an MCO-A classification [12] with 10 initial classes and a seven-fold symmetry. The MCO-A method converged to three different class, whose result is consistent with those published previously in [8, 12, 27, 28]. The central slices with each classification average resulting from the MCO-A classification are shown in Fig. 3, and class 1 is look-like the fitted volume of GroEL_14_, class 2 is associated with the fitted atomic model of GroEL_14_/ES_7_, class 3 is virtually less than the volume of GroEL_14_.

### Comparison of fine-grained subtomogram alignment accuracy to the baseline methods

We simulated 20 GroEL subtomograms with random rotation and translation of various SNRs under tilt range ±40^∘^ and ±60^∘^ respectively. We first compared our method with Chen’s approach [20] and Xu’s approach [21] to assess the subtomogram alignment accuracy against the noise-free reference volume, which was produced from the GroEL structure (PDB ID: 1KP8). The reference volume was low-pass filtered to 6nm resolution and was used as the starting reference for the alignment procedure.

We aligned the 20 simulated subtomograms with the reference volume using the three methods. The alignment accuracy was assessed using the constrained cross-correlation (CCC) defined in Section Parameter definitions. The resulting CCCs were compared using the t-test of pair-wise data between our method and the two baseline methods, where the data are assumed by normal distribution [29]. We also used non-parametric test without Gaussian assumption (Wilcoxon signed-rank test) to calculate *P*-value, and the results are similar to the t test (Supplementary Section 1).

As shown in Table 1, our method outperformed the two baseline methods using simulated subtomograms of SNR 0.03 and 0.003 under tilt range ±60^∘^.

**Table 1:** Alignment accuracy using *P*-value between our method and other methods under tilt range ±60^∘^

	SNR	C1	C2
	0.03	2.24E-05	0.01
	0.01	0.41	0.00
	0.003	1.01E-15	2.14E-13

We define C1, which is considered as *P*-value derived from the CCC values of our method minus the CCC values of the Xu’s method. We similarly define C2

The alignment accuracy comparison for subtomograms simulated with tilt angle range ±40^∘^ is shown in Table 2.

**Table 2:** Alignment accuracy using *P*-value between our method and other methods under tilt range ±40^∘^

	SNR	C3	C4
	0.03	0.04	1.32E-05
	0.01	0.19	0.02
	0.003	2.54E-05	3.08E-06

We define C3, which is considered as *P*-value derived from the CCC values of our method minus the CCC values of the Xu’s method. We similarly define C4

**Fig. 4:**
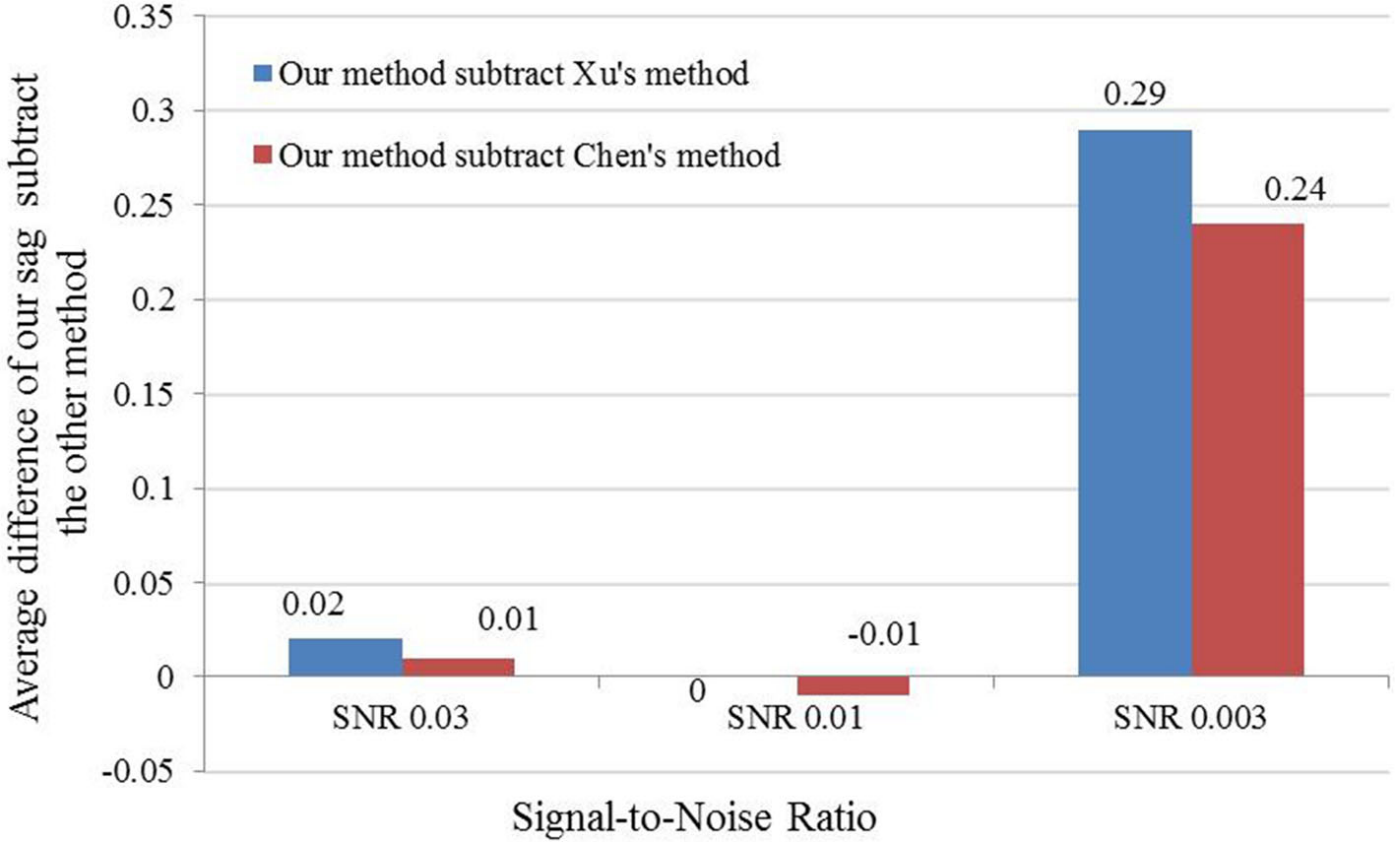
Comparison of methods under tilt range ±60^∘^. The mean value of difference of constrained cross-correlation obtained by our SAG fine-grained subtomogram alignment method and the other method under tilt range ±60^∘^

**Fig. 5:**
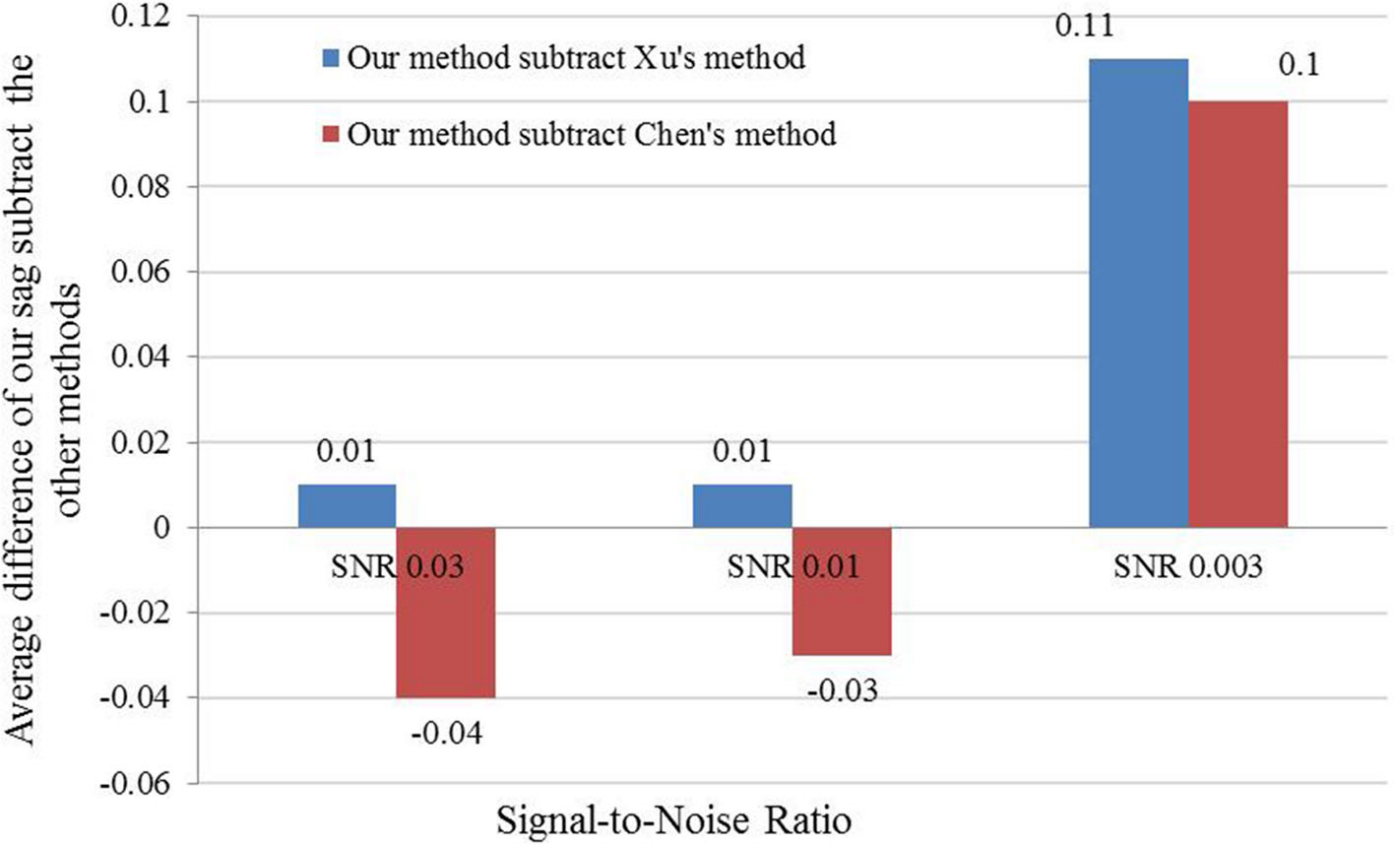
Comparison of methods under tilt range ±40^∘^. The mean value of difference of constrained cross-correlation obtained by our SAG fine-grained subtomogram alignment method and the other method under tilt range ±40^∘^

We note that although Chen’s method outperformed ours under some conditions, under a more realistic SNR 0.003 with different tilt angle ranges, our method has substantial improvement on the resulting CCC alignment accuracy (Figs. 4 and 5).

We also used 50 particles to evaluate subtomogram alignment accuracy under different conditions and compared the resolution value under the 0.143 criteria of FSC (Supplementary Section 2). This comparison proves that our method outperformed the two baseline methods using simulated subtomgrams of SNR 0.003 under tilt range ±60^∘^ and ±40^∘^.

### Computation time compared to other methods in subtomogram alignment

Next, we compared the computational time between our SAG fine-grained subtomogram alignment method and the Xu’s method and Chen’s method. For an objective and fair comparison, we implemented the three alignment method in Python and performed them on 20 simulated subtomogram of SNR 0.003 under tilt range ±60^∘^.

We used the original reference-free model as the initial reference for our algorithm. The most common Reference-free alignment rules are to use the subtomograms average in a random direction as an original reference [28]. The so-called no reference is not without any reference, but does not need a external reference, because external reference leads to reference bias. We recorded the running time of each method in obtaining the best resolution.

Every time the subtomogram alignment method converged, we got a resolution value. By defining the same convergence times, we evaluated which method can get the best resolution value with the shortest convergence times.

After each iteration, we got the subtomograms averaging and used FSC means to measure the resolutions, and then reported the running time for our SAG fine-grained subtomogram alignment method. Afterward, we repeated the protocol using Xu’s method and Chen’s method with an SNR of 0.003 conditions. Finally, we compared the resolutions of the average and the running time in three different subtomogram alignment methods.

**Fig. 6:**
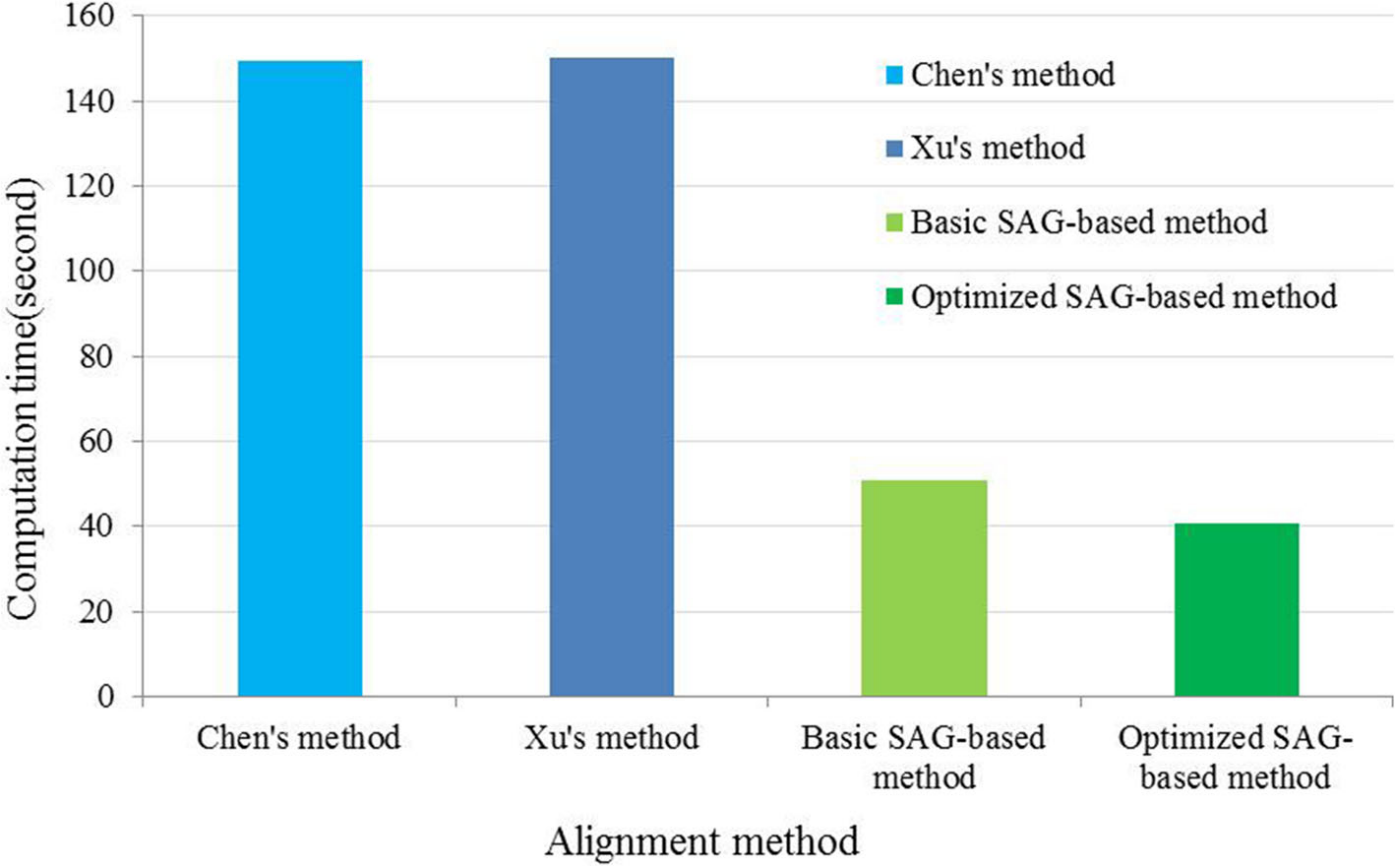
Computation time of different alignment method used once. The computation time of Chen’s alignment and Xu’s alignment method are shown by powder blue and blue respectively. The computation time of our basic and optimized SAG-based fine-grained subtomogram alignment are shown by light green and green respectively

The computation time cost of basic SAG fine-grained alignment method and optimized SAG fine-grained alignment method is 50.7 seconds and 40.5 seconds respectively, but Xu’s method and Chen’s method cost 150.2 seconds and 149.4 seconds respectively (Fig. 6). The computation time of different alignment method is the time for each alignment algorithm to be used once. Figure 6 depicts the computation time of different alignment algorithms (basic SAG fine-grained alignment method, optimized SAG fine-grained alignment method, Xu’s method and Chen’s method). We note that our SAG fine-grained alignment method is faster than Xu’s method and Chen’s method in the computation time.

Then we compared the elapsed time of getting the best resolution in three alignment methods. To get the best resolution, different alignment methods may run many times, for example, our optimized SAG-based fine-grained subtomogram alignment method got the best resolution (37.1Å) by iterating 14 times, Xu’s method got the best resolution (40.7Å) with 11 iterations and Chen’s method got the best resolution (39.7Å) with 13 iterations (Fig. 8).

**Fig. 7:**
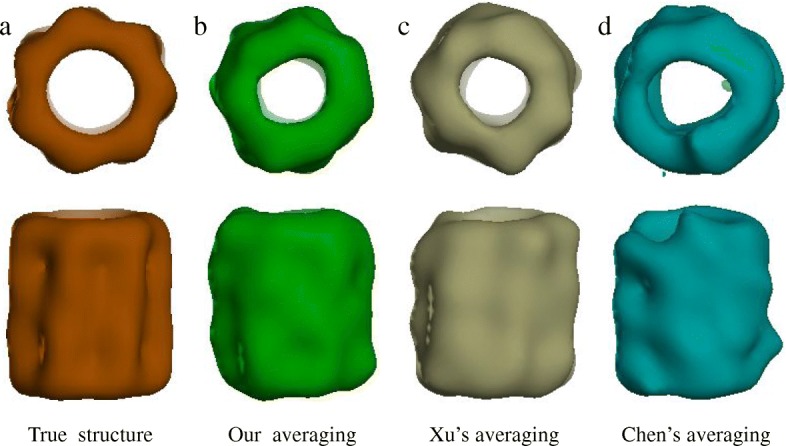
Average of three alignment method in SNR=0.003 under tilt range ±60^∘^. **a** Surface of effective GroEL structure (PDB ID: 1KP8) filtered to a resolution of 6nm. **b** Subtomograms average of our SAG fine-grained subtomogram alignment (resolution=37.1Å). **c** Subtomograms average of Xu’s alignment method (resolution=40.7Å). **d** Subtomograms average of Chen’s alignment method (resolution=39.7Å)

### Reference-free fine-grained alignment of subtomograms on simulated and experimental data set

We tested our SAG fine-grained alignment method and the two baseline alignment methods for subtomogram alignment without external reference. We first tested different alignment method on simulated subtomograms data set. Then we applied the three methods to the experimental GroEL subtomograms data set (Fig. 3) [8]. Subtomograms data sets were divided into odd and even data sets and aligned separately. The odd and even datasets were averaged separately. The normalised cross-correlation coefficient between the odd and even average density map over corresponding shells in Fourier space is measured by FSC to get many FSC values. Under the condition of FSC 0.143 that is “gold-standard” [30], the corresponding resolution values were calculated by many FSC and voxel values, and then the odd and even data sets were combined as the subtomograms average. The subtomograms average was used as a new reference and was low-pass filtered until the end of the cycle or the frequency did not meet the conditions.

**Fig. 8:**
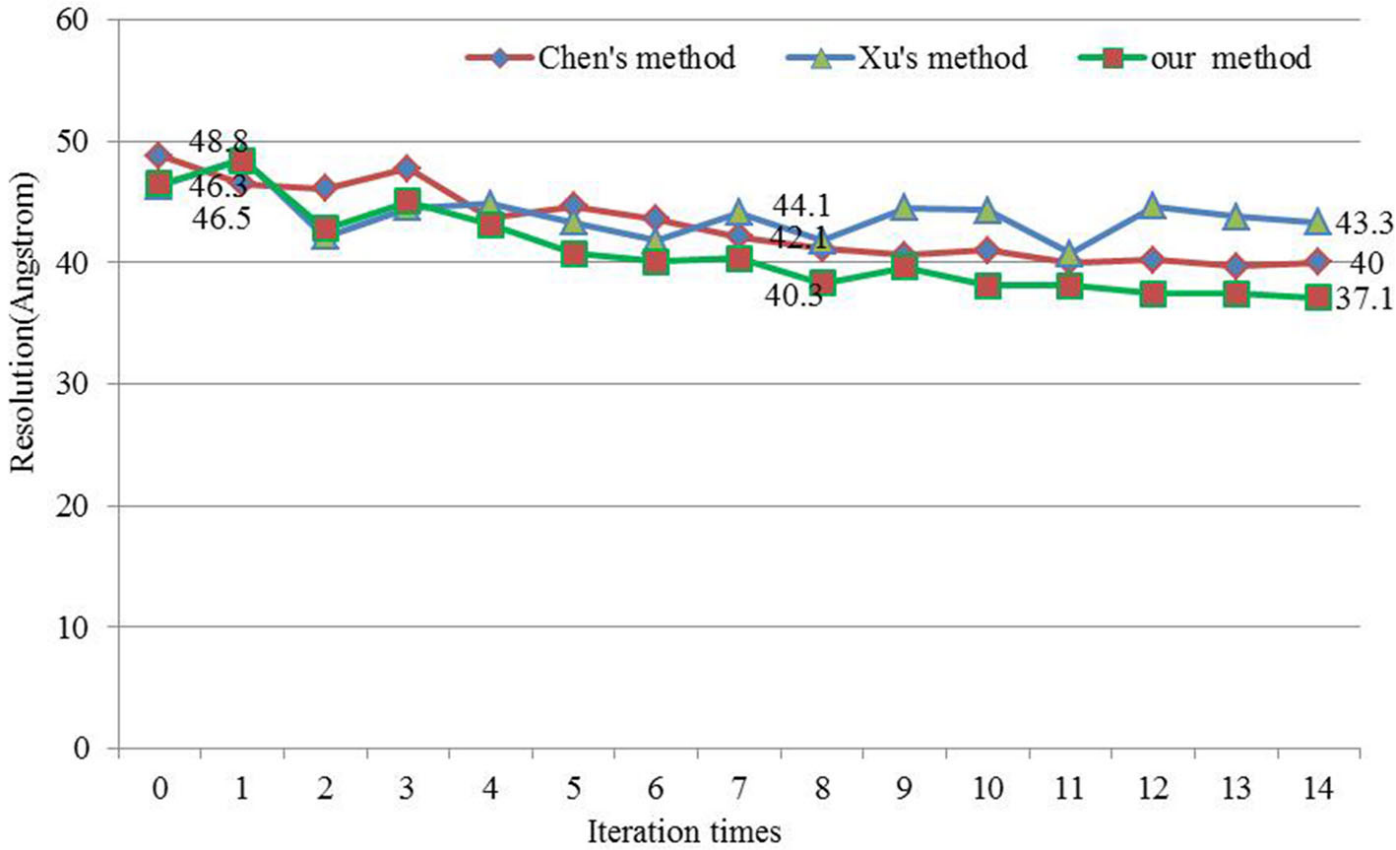
Iteration times of different alignment methods in obtaining the best resolution in SNR=0.003

We averaged the subtomograms after reference-free subtomogram alignment and computed their resolution curves. For simulated subtomograms dataset, our SAG fine-grained alignment method was applied for subtomogram alignment at SNR of 0.003 and tilt angle range ±60^∘^ (Figs. 7 and 8), and finally obtained the 37.1Å average resolution after 14 iterations according to gold-standard criteria of 0.143 FSC [30]. Applying Xu’s method and Chen’s method to subtomogram alignment respectively, the final average resolution (0.143 FSC criteria) was 40.7Å after 11 iterations and 39.7Å after 13 iterations respectively.

Our SAG fine-grained subtomogram alignment method can get better resolution than Xu’s alignment method, and slightly better than Chen’s alignment method. During the subtomogram averaging, we often need thousands of subtomograms and spend weeks to complete. Our SAG fine-grained subtomogram alignment method can reduce computational cost and get better resolution compared to the two baseline methods.

**Fig. 9:**
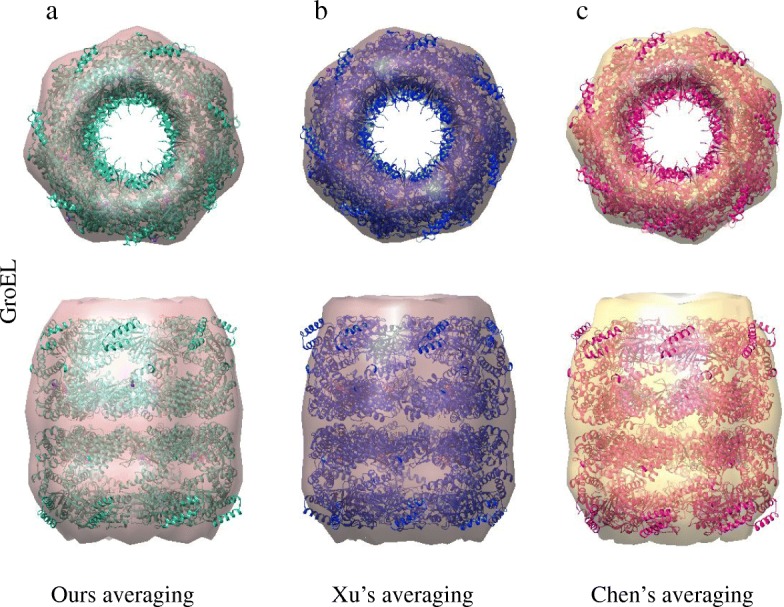
Averaging of experimental GroEL subtomograms. **a** The average of our method (red, final 25.1Å structure) fit into the GroEL_14_ atomic model (green). **b** The average of Xu’s method (gray, final 32.5Å structure) fit into the GroEL_14_ atomic model (blue). **c** The average of Chen’s method (yellow, final 27.9Å structure) fit into the GroEL_14_ atomic model (purple)

We then applied the three methods to an experimental GroEL subtomogram dataset (Fig. 3). Throughout our iterative alignment and averaging procedure, averaging of GroEL subtomograms transformed from a blurring structure to the barrel structure of the seven symmetry, resembling the true GroEL structure. According to the 0.143 criteria of FSC, the resolution of the final average was 25.1Å after 4 iterations (Fig. 9). In order to calculate the FSC resolution, all alignment methods were performed on the dataset divided into two independent halves.

Using Xu’s alignment method and Chen’s alignment method, the resolution of the final average (0.143 criteria) was 32.5Å after 9 iterations and 27.9Å after 12 iterations according to the FSC. Furthermore, we utilized the final average, which was acquired with different alignment methods, to fit atomic structures of complexes (PDB ID: 1KP8) in Fig. 9. From Fig. 9, the final average acquired by our SAG-based fine-grained alignment method is better than the final average acquired by Xu’s alignment method and Chen’s alignment method in subtomogram alignment procedure. Therefore, our SAG-based fine-grained alignment method outperforms Xu’s alignment method and Chen’s alignment method for subtomogram reference-free averaging.

We also added FSC curves for reference-free fine-grained alignment of subtomograms on simulated and experimental data set according to the 0.143 criterion (Supplementary Section 3).

## Discussion

In this article, we propose the stochastic average gradient (SAG) fine-grained alignment method by optimizing constrained dissimilarity scores. However, the original SAG algorithm was firstly applied to the two-dimensional matrix. So we designed two versions of 3D SAG-based fine-grained alignment method on subtomogram alignment procedure.

Since randomness introduces variance, standard stochastic gradient descent algorithm implements sublinear rates. Our SAG fine-grained subtomogram alignment method only selects the slice or the mini-batch slices along the x-axis in the experimental data in each iteration, maintains the memory of the latest gradient value calculated for each slice and the whole iteration produces a gradient of the subtomogram alignment. The size of mini-batch slices depends on the side length of subtomogram data. So our SAG fine-grained subtomogram alignment method has a linear convergence rate. On the other hand, by comparing the computational time between Algorithm 1 and 2, Algorithm 2 is faster than Algorithm 1, so Algorithm 2 is selected for fine-grained subtomogram alignment. But, Xu’s method and Chen’s method require the whole 3D volume to do the calculation in each iteration, and thus take more time. Compared to other methods, our method requires more temporary space in memory.

For the alignment accuracy comparison, Chen’s method performs better than our SAG fine-grained alignment method on SNR=0.03 and SNR=0.01 subtomograms under tilt range ±40^∘^, probably because Chen’s method searches for the best cross-correlation coefficient value between 3D cross-correlation matrix, which is accurate under higher SNR. However, our method is more robust to a more realistic low SNR setting of SNR 0.003.

Our SAG fine-grained alignment method uses MPI frame to calculate the score of dissimilarity in parallel for subtomogram alignment, however, using MPI is not easy to program and requires some experience, unlike multi-threading.

## Conclusion

Our SAG fine-grained subtomogram alignment method optimizes a constrained dissimilarity score in real space. It is obvious that our method is more accurate on subtomogram alignment and averaging at SNR=0.003 of tilt range ±60^∘^ and ±40^∘^. By comparing the elapsed time of different alignment method, our SAG fine-grained subtomogram alignment method is faster than Xu’s method and Chen’s method, and our method obtains better resolution, which is well validated on the simulated subtomograms datasets and experimental GroEL and GroEL/ES subtomograms datasets.

Additionally, we utilized a very efficient Message Passing Interface (MPI) frame parallel refinement alignment procedure, which is particularly designed to apply in parallel on multiple independent computers nodes connected by a network. MPI significantly accelerates the simultaneous refinement of multiple subtomogram alignment candidates set.

We will consider classification problems in the future and try to use new classification algorithms, not only including deep learning. In addition, we will continue to study subtomogram alignment. We will also test the new alignment algorithm with larger, updated subtomograms data sets.

Computational analysis of cryo-electron tomography is an emerging field due to its inherent content complexity and imaging limits [27, 31–37]. Our method serves as a useful step towards improved systematic recovery of macromolecular structures captured by such tomograms.

## Data Availability

Simulated GroEL (PDB ID:1KP8) subtomograms are available in Protein Data Bank. Experimental GroEL and GroEL/ES subtomograms datasets were used by Förster et al. [8]. The source code of the SAG-based fine-grained subtomogram alignment algorithm is available at https://github.com/xulabs/projects.
